# Controlled-release fertilizers increase sunflower yield by regulating soil nitrogen, photosynthesis, and root structure in arid regions

**DOI:** 10.3389/fpls.2025.1747095

**Published:** 2026-01-15

**Authors:** Wenhao Ren, Xianyue Li, Tingxi Liu, Ning Chen, Maoxin Xin, Qian Qi, Bin Liu

**Affiliations:** 1 State Key Laboratory of Water Engineering Ecology and Environment in Arid Area, Inner Mongolia Agricultural University, Hohhot, China; 2 Collaborative Innovation Center for Integrated Management of Water Resources and Water Environment in the Inner Mongolia Reaches of the Yellow River, Hohhot, China; 3 Research and Development of Efficient Water-saving Technology and Equipment and Research Engineering Center of Soil and Water Environment Effect in Arid Area of Inner Mongolia Autonomous Region, Hohhot, China; 4 Inner Mongolia Key Laboratory of Ecohydrology and High-Efficient Utilization of Water Resources, College of Water Conservancy and Civil Engineering, Inner Mongolia Agricultural University, Hohhot, China

**Keywords:** controlled-release fertilizer, photosynthesis, roots development, soil nitrogen, sunflower yield

## Abstract

**Background and aims:**

In arid irrigated systems, nitrogen supply often mismatches crop demand. This study assessed whether controlled-release fertilizers (CRF) better synchronizes nitrogen supply with sunflower demand than traditional nitrogen fertilizer (TNF), by comparing field treatments, quantified soil–root–plant responses, and identified the CRF rate that maximizes yields and nitrogen use efficiency (NUE).

**Methods:**

A three-year field experiment (2019–2021) was conducted in the Hetao Irrigation District, Bayannur, Inner Mongolia, China, using sunflower cultivar SH361. Treatments compared CRF and TNF at 135, 225, and 315 kg N/ha. Measurements included soil nitrate (0–100 cm), root traits (surface area density, dry weight), root sap production and sap nitrate, relative chlorophyll values, net photosynthetic rate, plant nitrogen uptake, and yield.

**Results:**

Relative to TNF, CRF significantly improved soil–root–plant N dynamics, increasing sunflower yield by 23.83%, plant NU by 8.17%, and NUE by 14.46%. CRF_225_ achieved the highest NUE while maintaining a yield statistically equivalent to CRF_315_, indicating that additional N input beyond 225 kg/ha conferred no yield benefit. Enhanced yield under CRF was strongly associated with higher soil nitrate availability, greater root activity, and increased photosynthesis.

**Conclusion:**

CRF improved nitrogen synchrony, yield, and NUE under arid irrigation. The 225 kg N/ha CRF rate provided the most favorable yield–efficiency balance, offering a practical management strategy for sustainable production in water-limited regions. By quantitatively linking CRF to soil–root–plant nitrogen coordination, this study advances understanding of nitrogen optimization in arid irrigated systems.

## 1 Introduction

Effective nitrogen management underpins crop productivity and maintaining agroecosystem sustainability (Maaz et al., 2021; Tei et al., 2020). Nitrogen is essential for nucleic acid and proteins and thus plant metabolism, growth and yield (Qiao et al., 2022; Wu et al., 2021a); deficiency depresses cell division and developmental, reducing productivity (Liu et al., 2018). Nitrogen availability is crucial in terrestrial ecosystems as a key determinant of primary productivity (Mason et al., 2022). Therefore, rational nitrogen input is critical for ensuring food security and sustaining agricultural output (Lam et al., 2022). Yet conventional nitrogen fertilizers is often used inefficiently, lowering plant uptake efficiency and increasing losses to water and air with economic and environmental costs (Lu et al., 2021; Penuelas and Sardans, 2022). These challenges highlight the need for fertilization strategies that increase nitrogen use efficiency by aligning fertilizer supply with crop demand over time, particularly in water-limited systems where timing strongly governs uptake and yield.

Despite extensive water diversion infrastructure (Liu et al., 2016), the Hetao Irrigation District in northwestern China remains constrained by an arid to semi-arid climate (Xiong et al., 2021). The region’s low and erratic rainfall severely limits soil moisture availability, restricting the range of crops capable of maintaining economic viability (Li et al., 2001; Wit et al., 2005). Sunflower is recognized for its high tolerance to drought and efficient water use, which supports its adaptability to limited moisture environments (Hussain et al., 2018; Zhang et al., 2022). Its robust root architecture facilitates deep water uptake, enhancing survival in arid soils (Jing et al., 2020; Zhang et al., 2021a). Maintaining soil nutrient supply is essential to support growth and metabolic function during stress. In particular, balanced fertilization remains a key strategy for improving oilseed yield and resilience (Ren et al., 2017; Seddaiu et al., 2016). Recent years have seen a growing dependence on nitrogen fertilizers to increase productivity (Garibaldi et al., 2011; Quemada and Lassaletta, 2024). Between 2016 and 2022, global N production rose from 116.72 to 122.56 million tons, while demand expanded from 105.15 to 111.59 million tons, highlighting the growing pressure to optimize nitrogen resource use (FAO, 2019). Under such water-limited environments, aligning nitrogen supply with crop demand is critical: when fertilizer inputs exceed uptake capacity, losses via nitrate leaching, denitrification, and ammonia volatilization increase (Naz and Sulaiman, 2016). whereas better temporal matching improves uptake, photosynthesis, and yield (Ashraf et al., 2019; Cameron et al., 2013; Shafqat et al., 2021). Balanced fertilization therefore remains essential for oilseed productivity and resilience (Corbeels et al., 1998; Mahpara, 2019; Waqar et al., 2022). Controlled-release fertilizers (CRF) provide a practical means to improve this synchrony by gradually releasing nitrogen in response to soil conditions, potentially reducing losses while sustaining supply compared with traditional nitrogen fertilizers (TNF) (Guo et al., 2021; ME Trenkel, 2021; Wang et al., 2018).

Recent studies indicate that adequate or stage-wise nitrogen application can not only increase leaf nitrogen content (Zhang et al., 2020) but also reprogram root development and activity (Xia et al., 2025). When nitrogen supply is greater or better timed, it can increase root surface area and biomass (Chen et al., 2020), thereby strengthening root acquisition of soil nitrogen (Wei et al., 2020), which in turn enhances leaf nitrogen status and photosynthesis (Mu and Chen, 2021) and ultimately promotes grain yield (Croce et al., 2024; Garcia et al., 2023). Recent advances also show that CRF can modify soil nitrogen dynamics and root development in ways that differ from TNF. For example, several studies have reported that CRF maintains higher soil nitrate availability during mid-growth stages (Li et al., 2021; Yang et al., 2021), promotes fine-root proliferation and root activity (Li et al., 2022; Ma et al., 2025), and enhances plant N uptake and biomass accumulation (Fan et al., 2025). However, despite these findings, the extent to which CRF improves the coordinated responses of soil nitrogen, root traits, photosynthesis, and yield under arid irrigated conditions remains poorly quantified. In the Hetao Irrigation District, nitrogen application rates for sunflower vary widely among farmers, and empirical over-fertilization remains common. This results in substantial residual nitrate in the soil profile and low NUE. Therefore, establishing a nitrogen-rate gradient is necessary to determine the relationships among soil nitrogen supply, plant nitrogen uptake, and yield, and to identify an agronomically optimal nitrogen input level. However, despite the increasing interest in CRF, quantitative field evidence from arid and semi-arid irrigated regions remains highly limited, especially regarding how CRF performs relative to TNF under controlled nitrogen input levels. In addition, although CRF is generally expected to synchronize nitrogen release with crop demand more effectively than TNF, it remains unclear under arid irrigated conditions whether CRF can outperform TNF across different nitrogen input levels, and at which nitrogen rate CRF can best enhance soil–root–plant synchrony to achieve the most favorable yield–efficiency balance. Consequently, directly comparing CRF with TNF under a controlled nitrogen gradient is essential for clarifying their relative effects on soil nitrate accumulation, plant nitrogen acquisition, photosynthesis, and yield.

Based on the above research gaps, we hypothesized that CRF would enhance soil–root–plant nitrogen coordination, increase nitrogen-use efficiency, and achieve a more favorable yield–efficiency balance than TNF under different nitrogen input rates. This was tested in a two-factor field experiment (fertilizer type: CRF vs. TNF; nitrogen rate: 135, 225, 315 kg N/ha) with measurements of soil nitrate, root traits, root-sap production and sap nitrate, leaf relative chlorophyll values, net photosynthetic rate, plant nitrogen uptake, and yield, and correlation analyses to examine soil–root–plant linkages consistent with the hypothesis. This three-year field experiment aimed to: (1) compare CRF with TNF across 135, 225, and 315 kg N/ha to test supply–demand synchrony and quantify responses in soil nitrate, root traits and root-sap indices, relative chlorophyll values, net photosynthetic rate, plant nitrogen uptake, and yield. (2) identify the nitrogen rate under CRF that achieves near-optimal yield with the highest nitrogen use efficiency, thereby defining a practical yield–efficiency trade-off. (3) elucidate soil–root–plant linkages by correlation analyses among soil nitrate, root traits/sap indices, photosynthesis, nitrogen uptake, and yield to clarify mechanisms underpinning CRF effects.

## 2 Materials and methods

### 2.1 Experimental site

**Figure 1:**
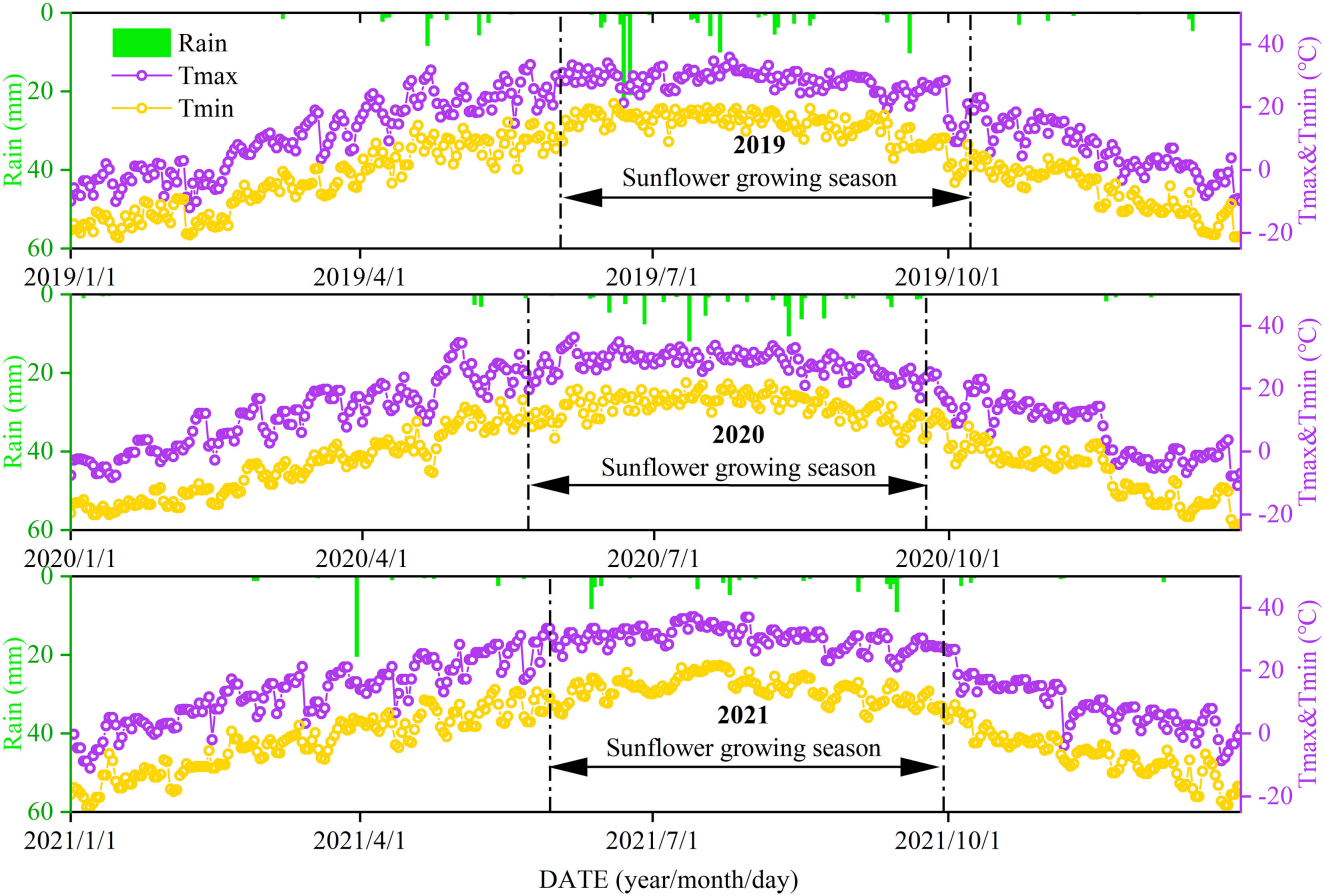
Precipitation (rain) and maximum (Tmax) and minimum (Tmin) temperatures recorded during the crop fertility periods from 2019 to 2021.

The field trials were carried out in 2019, 2020, and 2021 at an agricultural research site located in Ganzhaomiao Town, Linhe District, Bayannur City, Inner Mongolia, China (40°47′54″N, 107°16′42″E). The region experiences a temperate, semi-arid continental climate. Prior to the 2019 planting season, baseline assessments of soil physicochemical characteristics were performed. The soil was classified as sandy loam, suitable for sunflower cultivation. Key properties included: bulk density of 1.40 g/cm^3^, organic matter 6.19 g/kg, hydrolyzable nitrogen 34.43 mg/kg, available phosphorus 1.84 mg/kg, potassium 113.04 mg/kg, and a pH of 8.5. Meteorological observations during the growing seasons (2019–2021) were obtained via an automated weather station installed on-site (Onset Computer Inc., U30, Hobo, USA) (**Figure 1**).

### 2.2 Field management and experimental design

The experiment employed the local sunflower cultivar Xinjiang Sanrui SH361 (growth duration ~120 d) over three consecutive years (2019–2021). Tillage and land leveling were completed in May each year using a rotary tiller. Seeding occurred on June 2 (2019), May 22 (2020), and May 30 (2021), with corresponding harvests on October 8, September 24, and September 29, resulting in growth periods of 128, 125, and 123 days, respectively. A split-plot design with three replicates was adopted, where fertilizer type (CRF vs. TNF) was assigned to main plots and nitrogen application rate (135, 225, 315 kg/ha) to subplots, resulting in six treatments and 18 plots (each 24 m × 6 m, or 144 m^2^). CRF (N:P:K = 28:12:10, Tianjin Luyang Fertilizer Co., Ltd.) was applied once as a basal dressing. TNF plots received diammonium phosphate (N 18%, P_2_O_5_ 46%) as base fertilizer and urea (N 46%) as a top dressing at a 2:1 base-to-top ratio, ensuring that the total N input was consistent across CRF and TNF treatments. In addition, the application rates of P and K fertilizers were kept identical across all treatments to eliminate potential confounding effects of P and K on nitrogen response. Base fertilization was carried out prior to seeding on May 16 (2019), May 12 (2020), and May 10 (2021), corresponding to the pre-sowing stage. TNF topdressing was applied during the early bud initiation stage, on July 4 (2019), July 4 (2020), and July 12 (2021), when sunflower nitrogen demand increases sharply. Irrigation was provided via furrow method, targeting a depth of 120 mm on July 14 of each year. Additional field practices, including weeding and pest control, followed local agronomic standards.

### 2.3 Sampling and measurements

Soil nitrate concentration (SNC) was quantified using the semi-micro Kjeldahl method (Zhu et al., 2010). Composite soil samples were taken at 0–10, 10–20, 20–40, and 40–60 cm depths using an auger, with sampling every 10–15 days. For each sampling event, three discrete cores were collected from different points within the same plot and then homogenized to form one composite sample, ensuring consistent representation of soil nitrogen status. Sampling depth intervals and procedures were kept strictly consistent across all three experimental years (2019–2021). For each time point, triplicate cores from the same plot were collected, air-dried, and sieved (1 mm). To accurately capture nitrate availability and avoid disturbance-related fluctuations, soil sampling was conducted both before and after irrigation events and before and after fertilizer application. To extract available nitrogen, 25 mL of 2 mol/L KCl solution was added to 5 g of soil, stirred thoroughly, filtered, and analyzed for NO_3_-N content using a UV-1901 spectrophotometer (Beijing General Instrument Co., Ltd., China).

Nitrogen uptake (NU) was calculated as the total amount of nitrogen absorbed by the aboveground biomass at maturity (Equation 1). For each plot, five representative sunflower plants were oven-dried to a constant weight and ground to pass a 0.5-mm sieve. Nitrogen concentration was determined using the semi-micro Kjeldahl method. NU was then computed as:

(1) NU(kg/ha)=Aboveground biomass (kg/ha)×Plant N concentration (%)

This represents the total crop nitrogen uptake, rather than grain N accumulation alone.

Partial factor productivity of nitrogen (PFP) (Equation 2) was calculated to evaluate fertilizer productivity:

(2) $$PFP\;(kg/kg)=\frac{Grain\;yield\;(kg/ha)}{N\;applicationrate\;(kg/ha)}$$

Nitrogen use efficiency (NUE) (Equation 3) was defined following agronomic N-efficiency concepts, representing the yield produced per unit of nitrogen absorbed:

(3) $$NUE\;(kg/kg)=\frac{Grain\;yield\;(kg/ha)}{NU\;(kg/ha)}$$

This index reflects the crop’s internal utilization efficiency of absorbed nitrogen.

Sunflower roots were excavated via the profile method, then transferred to the lab. After initial sieving (3 mm), roots were isolated, gently washed to eliminate soil residues, and sealed for storage. Root surface area density was evaluated based on scanned images processed using WinRHIZO software. Root dry weight was determined after oven-drying samples at 80 °C to a constant mass. The formula for calculating the root surface area density (Equation 4) is as follows:

(4) $$RSD=\frac{Root\;surface\;area}{Soil\;volume}$$

To ensure measurement accuracy, the WinRHIZO system was calibrated before each scanning session using manufacturer-provided standard images, and root length/diameter calibration coefficients were applied following the software guidelines. Root sap was collected at four growth stages (seedling, bud, flowering, and maturity) using defatted cotton. On the first evening (18:00), sunflower stems were trimmed 10 cm above the soil surface, and cotton was secured around the stem to absorb the exudate overnight. At 6:00 the next morning, the cotton was retrieved, and sap yield was determined based on weight gain, assuming a density of 1.0 g/mL, to calculate production rate per unit time. Samples were then transferred into centrifuge tubes and spun at 20 min to separate the supernatant. A 1 mL aliquot of supernatant was diluted tenfold with distilled water and analyzed for NO_3_-N content using the semi-micro Kjeldahl method (Bremner, 1965), with quantification by UV-1901 spectrophotometer (Beijing General Instrument Co., Ltd., China). All sap measurements were corrected using blank cotton controls to account for background moisture absorption.

During the 2019–2021 field trials, three sunflower plants per plot were selected and labeled. Net photosynthetic rate (Pn, μmol CO_2_/m^2^/s) was measured at all four developmental stages using a portable photosynthesis system (Cpro T, Ecotech Ecological Technology, Beijing, China) on the uppermost fully expanded leaves between 9:00 and 11:00 a.m. under saturating light conditions, with three repeated measurements per plant. Relative chlorophyll values was determined via SPAD meter (Beijing, Jinkeli Electronics Technology, TYS-4N), with three readings per leaf averaged per plant. Additionally, at maturity, yield was determined by harvesting two central rows (24 m² per plot) to avoid border effects. Plants were threshed, and seed yield was measured and converted to kg/ha.

### 2.4 Statistical analysis

Microsoft Excel 2021 was used for preliminary data organization. A two-way analysis of variance (ANOVA) was conducted with fertilizer type (CRF vs. TNF) and nitrogen application rate (135, 225, and 315 kg N/ha) as fixed factors, including their interaction (type × rate). Analyses were performed separately for each year (2019–2021) due to inter-annual variability, and a combined analysis across years was also conducted. When significant effects were detected (p < 0.05), Tukey’s HSD test was used for multiple comparisons among treatments. Results are presented as means ± standard error (SE), and different letters indicate significant differences. All statistical analyses and figures were generated using OriginPro 2021.

## 3 Results

### 3.1 Effects of different fertilizer treatments on soil nitrate nitrogen

**Figure 2:**
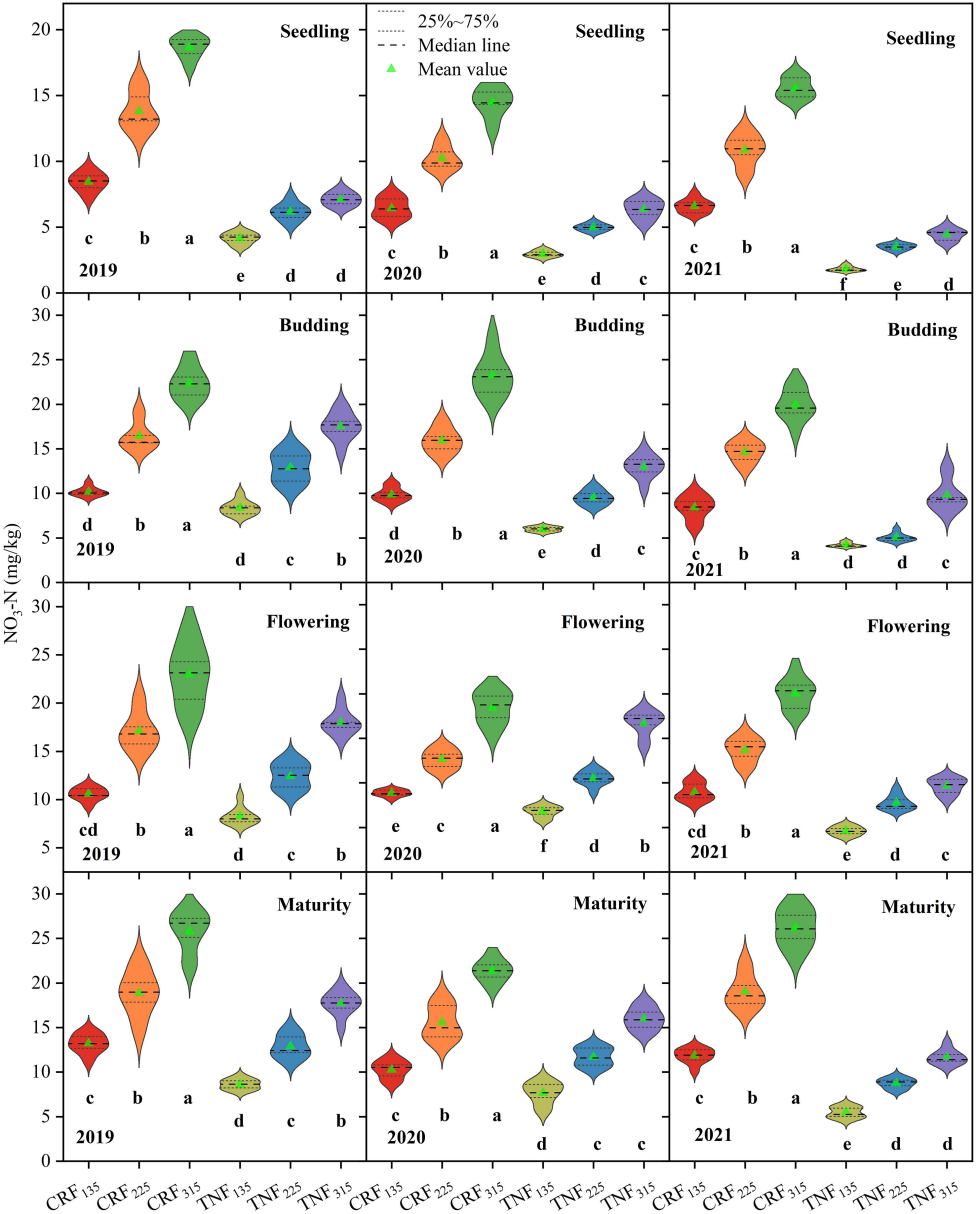
Soil NO_3_-N in sunflowers during the growing season under different nitrogen fertilizer types (CRF, TNF) and application rates (135, 225, 315 kg/ha) across 2019–2021. The violin plot represents the outcomes of all data processing, where wider distributions indicate higher data density and narrower distributions signify sparser data. Within each growth stage, significant differences between fertilization methods were evident in the violin plot, with distinct letters denoting differences at p < 0.05, as determined by Tukey’s HSD test. Different lowercase letters (e.g., a, b, c, and d) indicate significant differences between treatments at the p < 0.05 level.

The type of fertilizer and amount of N applied significantly affected SNC at different sunflower growth stages (**Figure 2**, **Table 1**). Two-way ANOVA indicated that both fertilizer type (T) and nitrogen rate (R) had significant effects across all stages (p < 0.01), while their interaction (T×R) was significant at seedling, budding, and maturity stages, but less consistent at flowering. Regardless of the fertilizer treatment, SNC increased from the seedling stage to the budding stage before declining at maturity, with higher N application rates consistently leading to greater SNC at each stage. Over the three-year experiment (2019–2021), compared to CRF_135_, the CRF_225_ and CRF_315_ treatments increased SNC by 52.45% and 116.03% during flowering and by 51.85% and 108.32%, respectively, at maturity. Similarly, compared to TNF_135_, TNF_225_ and TNF_315_ treatments increased SNC by 57.84% and 124.20% during flowering and by 54.31% and 72.94%, respectively, at maturity. Additionally, CRF treatments resulted in 48.08% higher SNC during flowering and 48.35% higher SNC at maturity compared to TNF.

**Table 1:** ANOVA for the main and interaction effects of fertilizer type (T) and rate (R) on Soil NO_3_-N under different nitrogen fertilizer types (CRF, TNF) and application rates (135, 225, 315 kg/ha) across 2019–2021.

Stage	ANOVA	2019	2020	2021	Total
Seeding	T	1180.43**	762.24**	1930.90**	916.92**
	R	280.75**	264.48**	358.02**	223.31**
	T×R	84.97**	44.29**	109.02**	57.15**
Budding	T	78.99**	372.74**	551.11**	242.91**
	R	258.37**	275.76**	213.55**	211.27**
	T×R	5.39**	27.39**	31.18**	14.77**
Flowering	T	69.11**	41.59**	515.47**	115.66**
	R	176.74**	341.41**	234.51**	194.40**
	T×R	3.09ns	0.17ns	35.31**	4.05*
Maturity	T	190.54**	125.68**	863.37**	356.89**
	R	194.27**	252.23**	281.42**	267.71**
	T×R	5.06*	5.15**	44.50**	14.68**

The symbols ‘**’ and ‘*’ indicate statistical significance for correlations with p-values less than 0.01 and 0.05, respectively, while “ns” denotes no significant differences. “Total” represents the sum of yields over the three years. For the interaction effect, mean values (n = 9) with different letters within the same column are significantly different at p < 0.05, as determined using Tukey’s HSD test.

### 3.2 Effect of different treatments on sunflower yield and nitrogen-use characteristics

**Figure 3:**
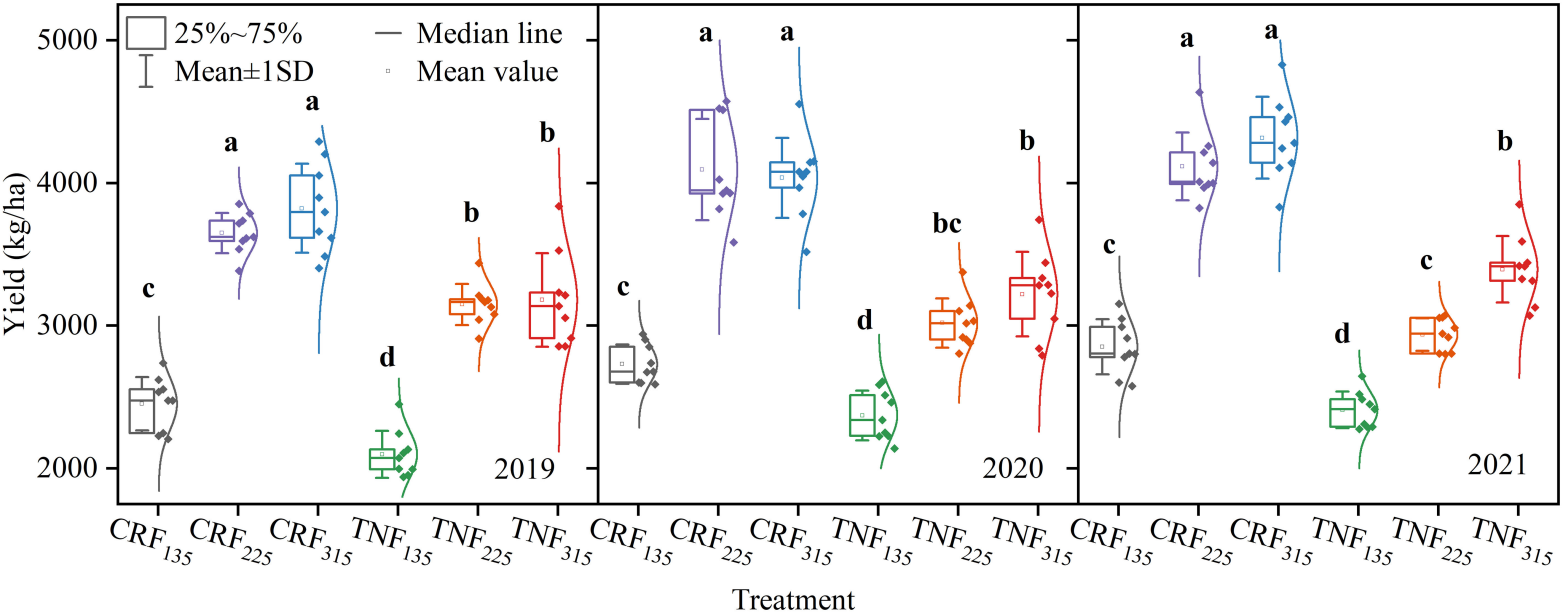
Sunflower yields under different nitrogen fertilizer types (CRF, TNF) and application rates (135, 225, 315 kg/ha) across 2019–2021. Different lowercase letters (e.g., a, b, c, and d) indicate significant differences between treatments at the p < 0.05 level.

Two-way ANOVA showed that both fertilizer type (T) and nitrogen rate (R) had significant effects on sunflower yield across all years (p < 0.01), while the interaction (T×R) was significant in 2020, 2021, and in the combined analysis, but not in 2019 (**Figure 3**, **Table 2**). At the same N application rates, CRF treatments increased yields by 23.83% compared to TNF, demonstrating that CRF could enhance sunflower yields and improve fertilizer utilization efficiency. Within the same fertilizer type, the yields increased with higher N application rates. Under CRF treatments, the CRF_225_ and CRF_315_ treatments increased the yields by 47.64% and 51.66%, respectively, compared to CRF_135_, with both increases being significant (p < 0.05). However, the 2.75% increase in yield from CRF_315_ compared to that from CRF_225_ was not significant, suggesting that CRF_225_ achieved a near-maximal yield with greater nitrogen-use efficiency. Under TNF treatment, TNF_225_ and TNF_315_ significantly increased the yields by 33.09% and 42.74%, respectively, compared to TNF_135_ (p < 0.05), while TNF_315_ increased the yields by 7.75% compared to TNF_225_ (p < 0.05).

**Table 2:** ANOVA for the main and interaction effects of fertilizer type (T) and rate (R) on sunflower yield under different nitrogen fertilizer types (CRF, TNF) and application rates (135, 225, 315 kg/ha) across 2019–2021.

ANOVA	2019	2020	2021	Total
T	65.77**	122.44**	223.08**	384.86**
R	162.83**	105.5**	166.6**	425.6**
T×R	1.82ns	9.55**	14.39**	20.36**

**p ≤ 0.01.

In addition, the results of NU, PFP, and NUE presented in **Table 3** further support the mechanisms underlying the observed yield differences among treatments. Overall, CRF consistently exhibited substantially higher NU across the three experimental years. Compared with TNF, CRF increased NU by 8.69%, 8.20%, and 7.62% at the 135, 225, and 315 kg/ha nitrogen rates, respectively, indicating that the controlled-release characteristics of CRF enhanced nitrogen absorption throughout the entire growing season. This trend is consistent with the rapid-release pattern of TNF, which makes nitrate more susceptible to leaching losses under irrigation.

**Table 3:** Nitrogen uptake (NU), partial factor productivity (PFP), and nitrogen use efficiency (NUE) of sunflower under different nitrogen fertilizer types (CRF, TNF) and application rates (135, 225, 315 kg/ha) across 2019–2021.

Treatment	2019			2020			2021		
	NU (kg/ha)	PFP (kg/kg)	NUE (kg/kg)	NU (kg/ha)	PFP (kg/kg)	NUE (kg/kg)	NU (kg/ha)	PFP (kg/kg)	NUE (kg/kg)
CRF_135_	175.60 ± 6.57bc	18.17 ± 1.39a	13.97 ± 1.07cd	223.22 ± 6.48c	20.23 ± 1.01a	12.23 ± 0.61cd	215.63 ± 6.48c	21.13 ± 1.44a	13.23 ± 0.9bc
CRF_225_	198.68 ± 1.17b	16.22 ± 0.63b	18.37 ± 0.71a	238.70 ± 4.54c	18.19 ± 1.57b	17.15 ± 1.48a	248.6 ± 13.62b	18.29 ± 1.05b	16.56 ± 0.95a
CRF_315_	263.95 ± 8.46a	12.14 ± 0.99d	14.48 ± 1.18c	285.52 ± 8.57a	12.81 ± 0.89c	14.13 ± 0.98b	278.5 ± 17.21a	13.71 ± 0.91c	15.5 ± 1.03a
TNF_135_	162.20 ± 7.23c	15.55 ± 1.21b	12.94 ± 1.01d	204.57 ± 4.77d	17.57 ± 1.29b	11.6 ± 0.85d	198.36 ± 14.97c	17.85 ± 0.95b	12.15 ± 0.65c
TNF_225_	194.88 ± 9.53b	13.99 ± 0.64c	16.16 ± 0.74b	224.72 ± 2.47c	13.42 ± 0.77c	13.43 ± 0.77bc	213.5 ± 11.92c	13.06 ± 0.51c	13.76 ± 0.53b
TNF_315_	247.81 ± 16.64a	10.1 ± 1.04e	12.83 ± 1.32d	264.53 ± 7.97b	10.23 ± 0.94d	12.18 ± 1.12cd	256.89 ± 9.85b	10.78 ± 0.74d	13.22 ± 0.91bc

Lowercase letters (e.g., a, b, c, and d) denote significant differences between groups. The significance level was established through statistical analysis and set at p < 0.05.

For NUE, CRF_225_ achieved values of 18.37, 17.15, and 16.56 kg/kg from 2019 to 2021, all of which were higher than those of TNF_225_ at the same N level. The NUE pattern closely matched the yield response, confirming that CRF_225_ provided the optimal balance between high productivity and efficient nitrogen utilization. Although CRF_315_ produced slightly higher yields than CRF_225_, its NUE declined by approximately 6.4%–21.2%, suggesting diminishing marginal returns as nitrogen inputs increased and crop N uptake approached saturation. Regarding PFP, CRF_135_—due to its lowest nitrogen input—consistently recorded the highest PFP values across all three years (e.g., 18.17 kg/kg in 2019). However, its absolute yield remained lower than that of the medium and high N treatments. This indicates that under the conditions of this study, CRF_225_ achieved the best trade-off between yield performance and nitrogen input efficiency, making it the most agronomically and economically advantageous nitrogen application strategy. The changes in these metrics were highly consistent with the yield results, further demonstrating that CRF enhances nitrogen uptake and NUE, thereby contributing to higher yield performance, whereas TNF is constrained by rapid nitrogen release and uneven utilization, resulting in lower NUE.

### 3.3 Effect of different treatments on sunflower root system

Different fertilizer types and N application rates significantly affected sunflower root surface area density (RSD) and root dry weight (RDW) at various growth stages (**Table 4**). The CRF_315_ treatment achieved the highest RSD (3.53 cm^2^/cm^3^) and RDW (14.70 g/plant) during the flowering stage. Over the three-year experiment, compared to CRF_135_, the CRF_225_ and CRF_315_ treatments increased RSD and RDW by 10.11% and 9.48%, and 17.25% and 12.77%, respectively, during the flowering stage. In contrast, at maturity, these increases were 18.66% and 8.51% and 25.96% and 12.02%, respectively. Similarly, compared with TNF_135_, the TNF_225_ and TNF_315_ treatments increased RSD and RDW by 5.91% and 9.34% and 11.39% and 16.71%, respectively, during the flowering stage and by 8.66% and 10.73% and 17.12% and 15.82%, respectively, at maturity. Overall, CRF treatments were more beneficial for sunflower root growth and development than TNF, increasing RSD and RDW by 13.19% and 9.77% during flowering and by 16.28% and 19.64%, respectively, at maturity.

**Table 4:** Root surface area density (RSD) and root dry weight (RDW) of sunflowers during the growing season under different nitrogen fertilizer types (CRF, TNF) and application rates (135, 225, 315 kg/ha) across 2019–2021.

Treatments		RSD (cm^2^/cm^3^)			RDW (g/plant)		
		2019	2020	2021	2019	2020	2021
Seeding stage	CRF_135_	1.06 ± 0.05cd	0.83 ± 0.05c	0.93 ± 0.06b	2.02 ± 0.08b	2.2 ± 0.11d	2.38 ± 0.16d
	CRF_225_	1.19 ± 0.05ab	0.94 ± 0.04b	1.08 ± 0.11a	2.53 ± 0.1a	3.21 ± 0.18b	3.23 ± 0.33b
	CRF_315_	1.26 ± 0.09a	1.01 ± 0.03a	1.15 ± 0.05a	2.63 ± 0.14a	3.87 ± 0.14a	3.6 ± 0.36a
	TNF_135_	0.81 ± 0.03e	0.76 ± 0.04d	0.79 ± 0.05c	1.35 ± 0.05d	1.9 ± 0.09e	1.79 ± 0.21e
	TNF_225_	1 ± 0.03d	0.79 ± 0.03cd	0.91 ± 0.08b	1.65 ± 0.09c	2.92 ± 0.11c	2.55 ± 0.23cd
	TNF_315_	1.13 ± 0.06bc	0.74 ± 0.05d	0.95 ± 0.05b	1.9 ± 0.06b	3.21 ± 0.12b	2.85 ± 0.18c
	T	159.51**	253.36**	81.06**	911.79**	143.64**	93.86**
	R	102.35**	24.53**	32.69**	189.16**	635.69**	94.85**
	T*R	5.54**	30.08**	0.79ns	5.9**	11.75**	0.40ns
Budding stage	CRF_135_	3.43 ± 0.11c	3.01 ± 0.09b	3.23 ± 0.23bc	11.76 ± 0.25c	11.6 ± 0.42b	13.19 ± 1.12bc
	CRF_225_	3.69 ± 0.22b	3.39 ± 0.12a	3.6 ± 0.18ab	12.75 ± 0.54b	13 ± 0.66a	14.37 ± 0.85ab
	CRF_315_	3.97 ± 0.16a	3.59 ± 0.16a	3.86 ± 0.45a	14.36 ± 0.57a	13.63 ± 0.5a	15.73 ± 1.24a
	TNF_135_	3.13 ± 0.17d	2.75 ± 0.17c	2.98 ± 0.19c	10.22 ± 0.45d	10.19 ± 0.39c	11.33 ± 0.74d
	TNF_225_	3.34 ± 0.12cd	3 ± 0.2b	3.17 ± 0.25c	11.33 ± 0.52c	11.5 ± 0.47b	12.73 ± 1.06cd
	TNF_315_	3.45 ± 0.13c	3.15 ± 0.18b	3.32 ± 0.17bc	12.47 ± 0.59b	11.97 ± 0.47b	13.44 ± 1.23bc
	T	84.00**	68.36**	31.81**	140.97**	128.7**	45.03**
	R	34.36**	43.82**	15.19**	106.07**	71.35**	21.98**
	T*R	2.59ns	1.56ns	1.33ns	1.05ns	0.31ns	0.44ns
Flowering stage	CRF_135_	2.99 ± 0.13bc	3.02 ± 0.16b	3.03 ± 0.24bcd	12.52 ± 0.43bc	12.69 ± 0.49c	13.89 ± 0.79bc
	CRF_225_	3.35 ± 0.16a	3.3 ± 0.17a	3.31 ± 0.24ab	13.83 ± 0.5a	13.69 ± 0.73ab	15.29 ± 0.6ab
	CRF_315_	3.53 ± 0.2a	3.5 ± 0.14a	3.57 ± 0.37a	14.4 ± 0.56a	13.91 ± 0.56a	15.78 ± 2.04a
	TNF_135_	2.84 ± 0.17c	2.64 ± 0.15c	2.76 ± 0.16d	10.71 ± 0.54d	11.99 ± 0.48c	12.61 ± 1.11c
	TNF_225_	2.91 ± 0.17bc	2.91 ± 0.1b	2.89 ± 0.2cd	11.99 ± 0.48c	12.83 ± 0.83bc	13.74 ± 1.34bc
	TNF_315_	3.07 ± 0.13b	3 ± 0.15b	3.1 ± 0.16bc	12.86 ± 0.63b	13.66 ± 0.49ab	14.64 ± 1.1ab
	T	63.44**	112.64**	34.89**	145.32**	13.13**	15.22**
	R	26.48**	38.14**	15.20**	67.35**	25.79**	11.42**
	T*R	5.13**	0.86ns	0.87ns	0.44ns	1.16ns	0.12ns
Maturity stage	CRF_135_	2.2 ± 0.09c	2.03 ± 0.13c	2.13 ± 0.09bc	11.69 ± 0.58b	11.88 ± 0.7d	13.02 ± 1.1b
	CRF_225_	2.55 ± 0.14a	2.45 ± 0.13b	2.54 ± 0.14a	13.48 ± 0.66a	13.09 ± 0.48ab	14.63 ± 0.88a
	CRF_315_	2.68 ± 0.05a	2.63 ± 0.16a	2.69 ± 0.29a	13.93 ± 0.88a	13.88 ± 0.66a	15.69 ± 1.53a
	TNF_135_	2.01 ± 0.11d	1.83 ± 0.07d	1.94 ± 0.16c	9.6 ± 0.54d	10.33 ± 0.39e	11.21 ± 0.81c
	TNF_225_	2.14 ± 0.12cd	2.01 ± 0.1c	2.11 ± 0.16bc	10.16 ± 0.55cd	12.05 ± 0.49cd	12.29 ± 0.89bc
	TNF_315_	2.39 ± 0.15b	2.13 ± 0.1c	2.24 ± 0.11b	10.55 ± 0.46c	12.72 ± 0.72bc	12.81 ± 1.21b
	T	89.91**	135.49**	57.46**	294.01**	61.24**	61.13**
	R	63.74**	66.96**	30.03**	31.35**	65.59**	17.37**
	T*R	4.18*	7.59**	3.07ns	6.05**	0.92ns	1.05ns

Lowercase letters (e.g., a, b, c, and d) denote significant differences between groups. The significance level determined through statistical analysis was set at p < 0.05.

**Figure 4:**
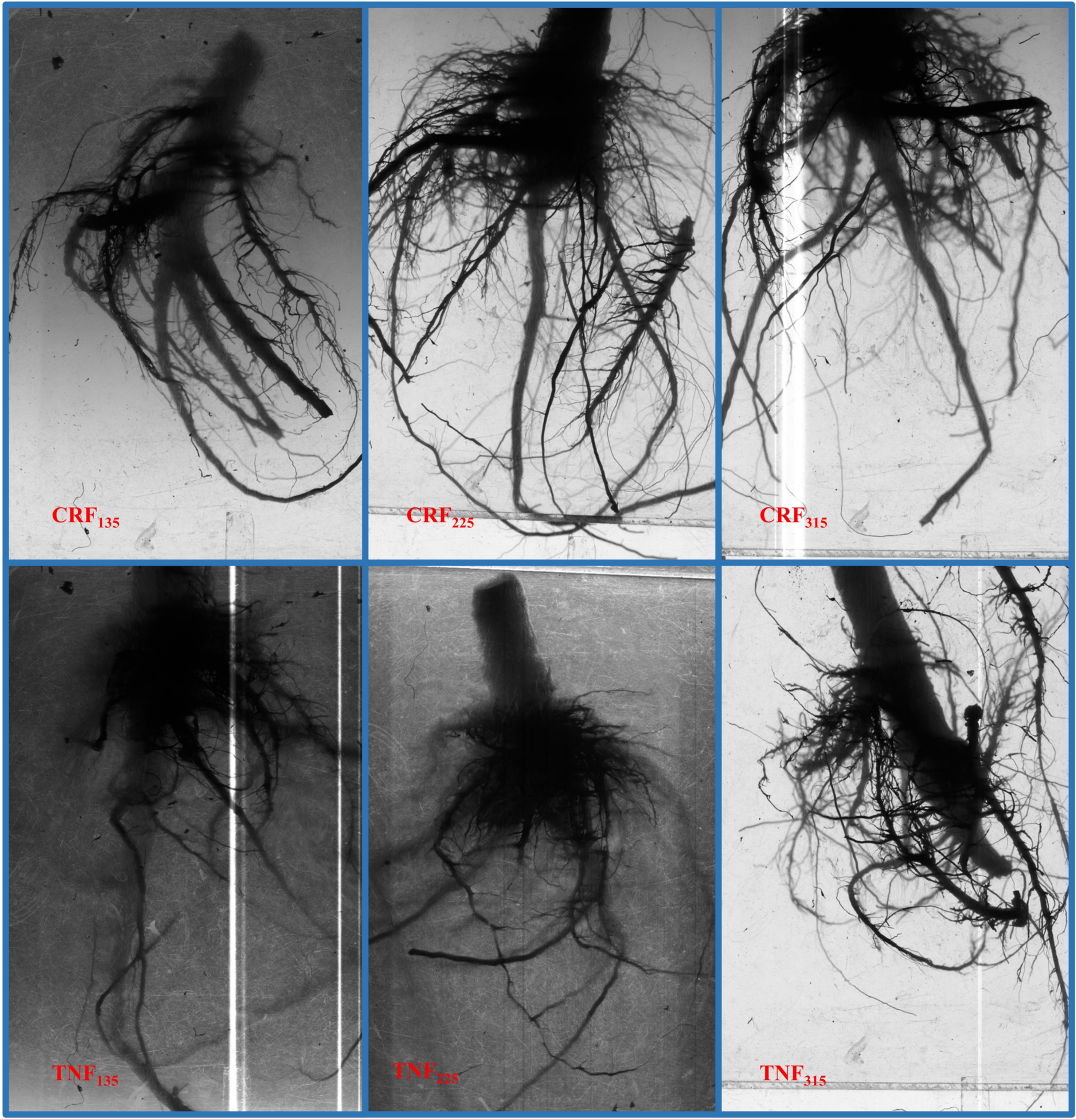
Scanned images of sunflower roots at the budding stage after under different nitrogen fertilizer types (CRF, TNF) and application rates (135, 225, 315 kg/ha) across 2019–2021.

Different fertilizer types and application rates significantly influenced sunflower root development, as observed in the root scanner images (**Figure 4**). CRF treatment resulted in longer primary roots, with the lateral roots branching out after the main root penetrated deep into the soil. In contrast, the TNF treatments prioritized lateral root development over primary roots, leading to more lateral growth concentrated in shallow soil layers. Regardless of fertilizer type, increasing N application promoted overall root system growth.

Throughout the sunflower growth cycle, both the CRF and TNF treatments exhibited an increase in root sap production rate (RSPR) across all stages, including seedling, budding, flowering, and maturity, as the N application increased (**Table 5**). At the seedling stage, although RSPR exhibited a slight increase with nitrogen application, no statistically significant differences were detected between CRF and TNF treatments (p > 0.05). Across the three-year study, CRF_315_ resulted in RSPR values that were, on average, 3.71% and 18.79% higher than those recorded under CRF_225_ and CRF_135_, respectively. In contrast, TNF_315_ led to increases of 3.80% and 20.50% relative to TNF_225_ and TNF_135_. RSPR peaked during the budding phase, where CRF-treated plants surpassed TNF by 5.24% (0.088 mL/h/root). Furthermore, at the flowering and maturation periods, CRF consistently maintained a 6.70% advantage in RSPR over TNF.

**Table 5:** Root sap production rate (RSPR) and NO_3_-N concentration in root sap (RSN) at different sunflower growth stages under different nitrogen fertilizer types (CRF, TNF) and application rates (135, 225, 315 kg/ha) across 2019–2021.

Treatments		RSPR (ml/h/root)			RSN (mg/kg)		
		2019	2020	2021	2019	2020	2021
Seeding stage	CRF_135_	1.38 ± 0.1bc	1.4 ± 0.07ab	1.57 ± 0.11bc	253.64 ± 17.45c	286.54 ± 18.79c	299.8 ± 21.35c
	CRF_225_	1.52 ± 0.09ab	1.58 ± 0.14ab	1.75 ± 0.14ab	375.8 ± 27.61b	396.46 ± 26.26b	421.65 ± 36.79b
	CRF_315_	1.57 ± 0.09a	1.63 ± 0.25a	1.8 ± 0.17a	435.6 ± 28.18a	432.55 ± 31.85a	472.27 ± 35.17a
	TNF_135_	1.32 ± 0.09c	1.37 ± 0.08b	1.48 ± 0.16c	269.6 ± 25.44c	264.25 ± 17.49c	296.29 ± 24.46c
	TNF_225_	1.51 ± 0.11ab	1.54 ± 0.21ab	1.72 ± 0.16ab	365.32 ± 29.31b	375.39 ± 25.86b	410.72 ± 40.35b
	TNF_315_	1.56 ± 0.16a	1.61 ± 0.16a	1.76 ± 0.06ab	389.5 ± 28.07b	396.26 ± 27.92b	431.38 ± 42.41ab
	T	1.00ns	0.55ns	2.01ns	3.57ns	14.98**	3.90ns
	R	19.94**	9.87**	18.00**	157.71**	152.78**	98.97**
	T*R	0.44ns	0.01ns	0.22ns	6.3**	0.51**	1.5**
Budding stage	CRF_135_	2.52 ± 0.11cd	2.59 ± 0.24cd	2.82 ± 0.22bc	645.3 ± 51.37c	612.35 ± 36.09c	694.22 ± 82.51c
	CRF_225_	2.96 ± 0.22ab	3.05 ± 0.23ab	3.35 ± 0.29a	803.6 ± 62.91b	817.47 ± 101.21ab	889.97 ± 80.76ab
	CRF_315_	3.07 ± 0.26a	3.22 ± 0.2a	3.45 ± 0.2a	897.36 ± 37.11a	889.25 ± 41a	988.89 ± 83.23a
	TNF_135_	2.29 ± 0.15d	2.31 ± 0.25d	2.57 ± 0.14c	574.5 ± 44.38c	602.32 ± 39.44c	651.37 ± 63.33c
	TNF_225_	2.76 ± 0.22bc	2.81 ± 0.15bc	3.13 ± 0.18ab	745.9 ± 54.41b	785.86 ± 67.88b	836.34 ± 60.35b
	TNF_315_	2.85 ± 0.23ab	2.92 ± 0.35abc	3.23 ± 0.33a	795.4 ± 55.89b	812.35 ± 47.23ab	890.69 ± 90.5ab
	T	15.05**	16.49**	13.11**	29.82**	5.85*	9.45**
	R	37.22**	31.56**	39.63**	99.08**	82.8**	56.54**
	T*R	0.03ns	0.05ns	0.02ns	0.87ns	1.46ns	0.64ns
Flowering stage	CRF_135_	1.55 ± 0.18b	1.6 ± 0.12bc	1.74 ± 0.14bc	89.5 ± 6.3d	102.35 ± 8.64d	106.38 ± 5.88d
	CRF_225_	1.84 ± 0.12a	1.88 ± 0.17a	2.08 ± 0.15a	156.3 ± 13.84b	157.74 ± 11.27b	171.62 ± 9.21b
	CRF_315_	1.9 ± 0.13a	1.92 ± 0.28a	2.14 ± 0.22a	193.5 ± 17.35a	180.5 ± 15.69a	207.01 ± 10.78a
	TNF_135_	1.43 ± 0.09b	1.48 ± 0.07c	1.62 ± 0.17c	103.5 ± 6.58d	94.32 ± 7.76d	109.1 ± 5.2d
	TNF_225_	1.73 ± 0.16a	1.78 ± 0.11ab	1.96 ± 0.23ab	139.6 ± 11c	134.7 ± 7.6c	148.26 ± 10.53c
	TNF_315_	1.86 ± 0.06a	1.92 ± 0.11a	2.09 ± 0.28a	141.23 ± 10.91bc	150.26 ± 12.07b	160.32 ± 6.42b
	T	6.69*	3.3ns	2.96ns	33.35**	47.54**	98.36**
	R	45.39**	28.27**	22.62**	177.62**	180.99**	392.72**
	T*R	0.60ns	0.74ns	0.17ns	36.42**	4.87*	39.75**
Maturity stage	CRF_135_	0.71 ± 0.07cd	0.8 ± 0.04bc	0.85 ± 0.06cd	83.2 ± 6.85c	77.63 ± 7.12d	88.05 ± 4d
	CRF_225_	0.85 ± 0.06ab	0.88 ± 0.08ab	0.97 ± 0.09ab	112.35 ± 9.3b	123.37 ± 7.68b	129.06 ± 15.49b
	CRF_315_	0.9 ± 0.08a	0.94 ± 0.09a	1.03 ± 0.07a	130.12 ± 8.38a	142.56 ± 12.05a	149.43 ± 11.3a
	TNF_135_	0.69 ± 0.05d	0.74 ± 0.06c	0.8 ± 0.11d	55.42 ± 2.91d	60.5 ± 7.47e	63.18 ± 4.32e
	TNF_225_	0.8 ± 0.11bc	0.82 ± 0.08bc	0.9 ± 0.05bcd	92.45 ± 5.43c	99.9 ± 11.35c	105.02 ± 8.67c
	TNF_315_	0.81 ± 0.05ab	0.85 ± 0.06ab	0.92 ± 0.06bc	105.68 ± 9.22b	112.5 ± 5.68b	119.45 ± 9.16b
	T	7.36**	15.2**	13.63**	143.36**	95.25**	99.81**
	R	23.03**	15.62**	18.48**	203.75**	209.17**	175.74**
	T*R	1.13ns	0.35ns	0.72ns	1.29ns	2.39ns	0.50ns

Lowercase letters (e.g., a, b, c, and d) denote significant differences between groups. The significance level was established through statistical analysis and set at p < 0.05.

NO_3_-N concentration in root sap (RSN) is a vital indicator of plant nitrogen uptake. Throughout the three-year trial, RSN values were generally elevated under CRF compared to TNF, with more distinct differences emerging at higher nitrogen input levels (**Table 5**). During the budding stage, CRF treatments resulted in a 15.54% increase (20.40 mg/kg) in RSN over TNF. Specifically, CRF_315_ boosted RSN by 13.43% and 61.83% compared to CRF_225_ and CRF_135_, while TNF_315_ showed corresponding increases of 7.33% and 50.66% versus TNF_225_ and TNF_135_. In the flowering and maturation phases, CRF maintained a 21.35% RSN advantage (22.52 mg/kg) relative to TNF. Notably, the highest RSN occurred at the budding stage (141.45 mg/kg), tapering to 102.77 mg/kg by maturity.

**Figure 5:**
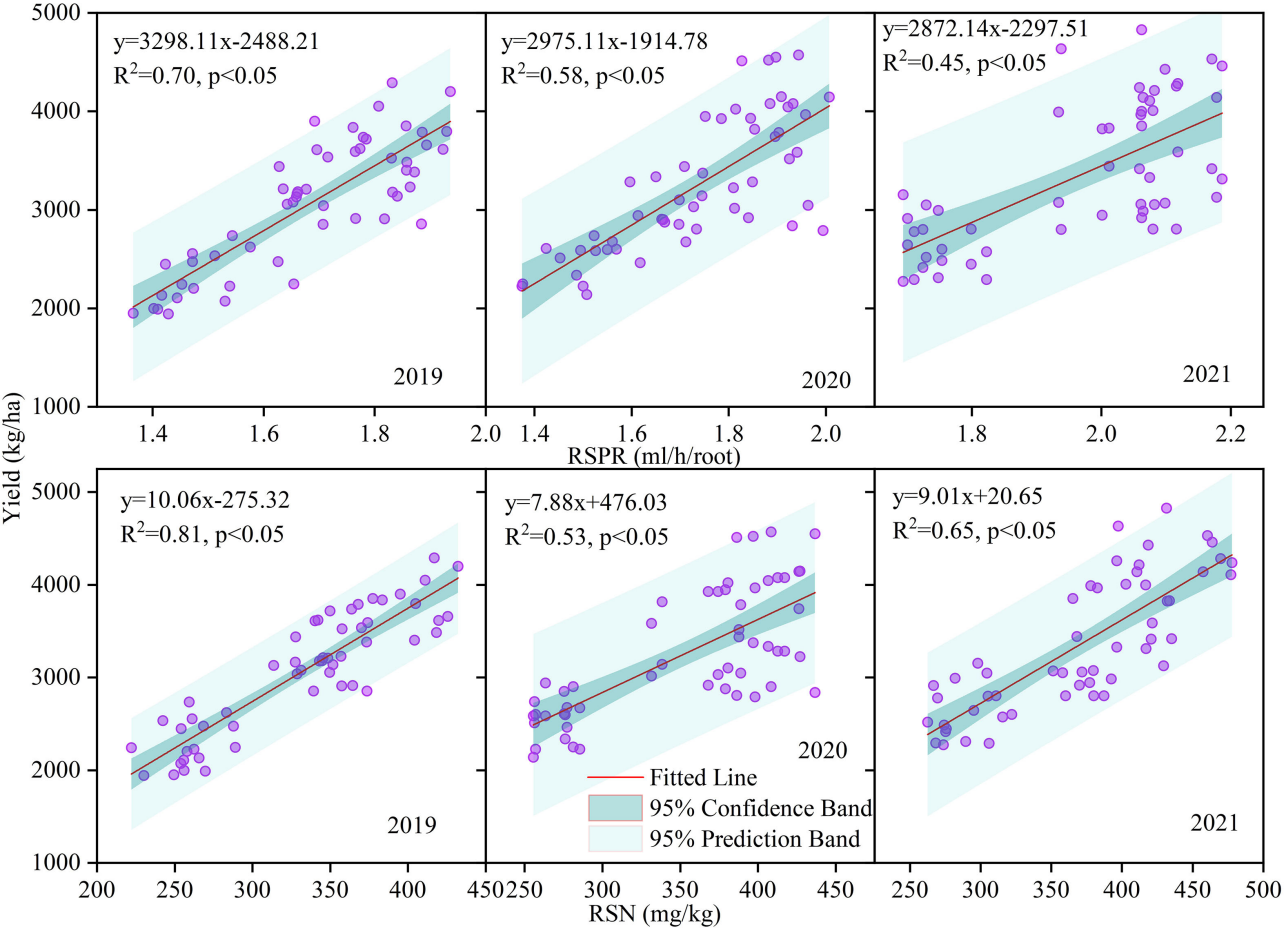
Correlations between grain yield and root sap production rate (RSPR) and nitrate-nitrogen concentration in root sap (RSN) under different nitrogen fertilizer types (CRF and TNF) and application rates (135, 225, 315 kg/ha) across 2019–2021.

From 2019 to 2021, grain yield exhibited a positive association with both RSN and RSPR under varied nitrogen strategies (**Figure 5**). Yield performance improved with higher RSPR (R^2^ = 0.70–0.45, p < 0.05) and elevated RSN levels (R^2^ = 0.81–0.53, p < 0.05), confirming significant linear trends.

### 3.4 Effect of different fertilizer treatments on Net photosynthetic rate and relative chlorophyll values

**Figure 6:**
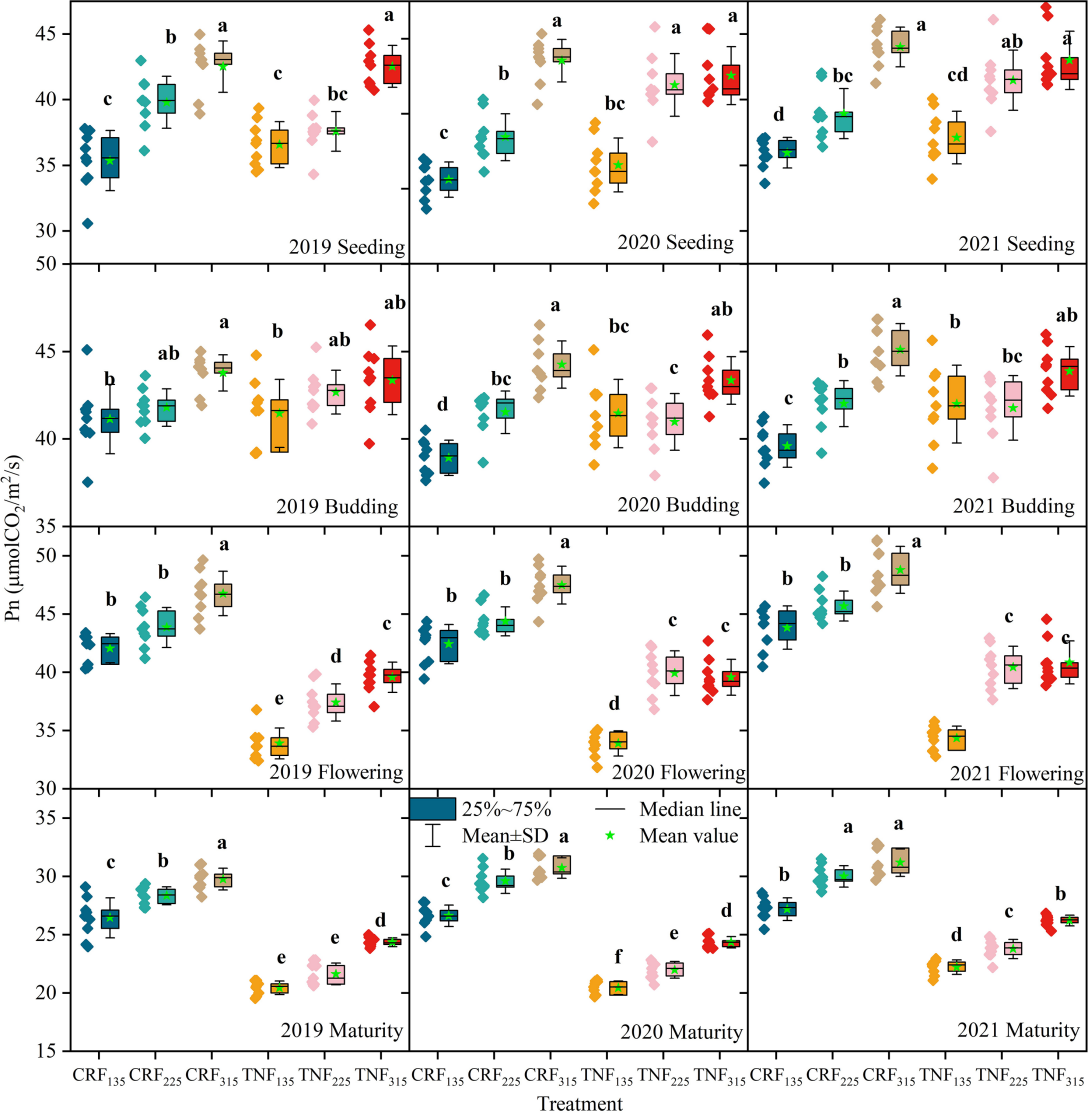
Net photosynthetic rate (Pn, μmol CO_2_/m^2^/s) of sunflowers during the growing season under different nitrogen fertilizer types (CRF, TNF) and application rates (135, 225, 315 kg/ha) across 2019–2021. Means (n = 9) followed by different letters within a column differ significantly at p < 0.05, according to Tukey’s HSD.

**Figure 7:**
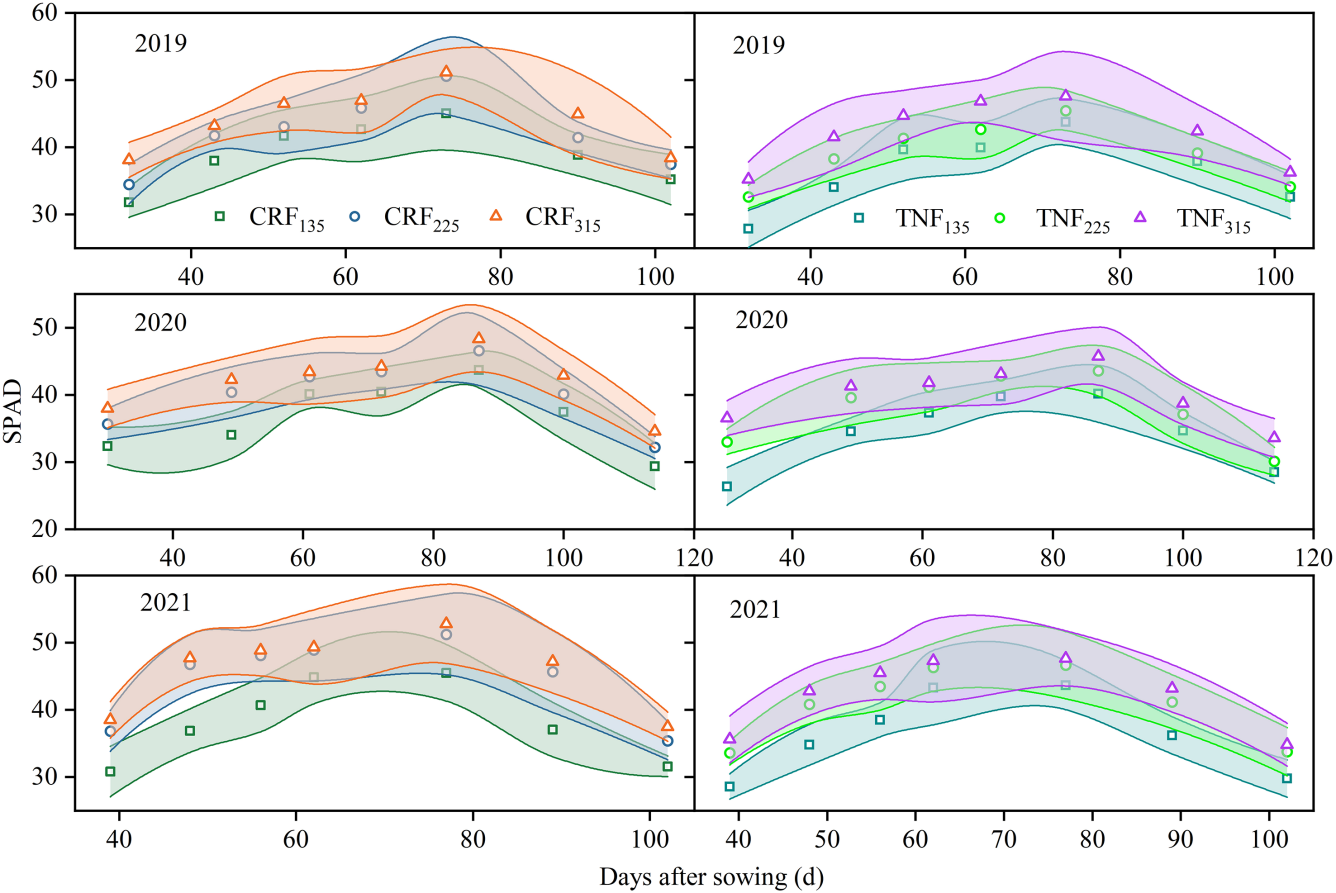
Relative chlorophyll values of sunflowers during the growing season under different nitrogen fertilizer types (CRF, TNF) and application rates (135, 225, 315 kg/ha) across 2019–2021. Relative chlorophyll values (SPAD readings).

Between 2019 and 2021, variations in net photosynthetic rate (Pn) and relative chlorophyll values of sunflowers were significantly influenced by fertilizer types and nitrogen application levels (**Figures 6**, **7**; **Tables 6**, **7**). Two-way ANOVA indicated that nitrogen rate (R) consistently exerted a significant effect across all growth stages (p < 0.01). Fertilizer type (T) was significant mainly during flowering and maturity, while the T×R interaction was significant at selected stages only. Under CRF treatments, both indices gradually increased with increasing N application. In the seedling stage, compared to CRF_135_, CRF_225_ and CRF_315_ increased Pn by 9.58% and 21.64% and relative chlorophyll values by 12.72% and 20.61%, respectively. By the budding stage, these increases were 4.80% and 9.33% for CRF_225_, and 11.34% and 13.23% for CRF_315_, respectively. During the seedling and budding stages, the relative chlorophyll values for CRF_225_ were slightly higher than those for TNF_225_, exceeding them by 7.81% and 6.23%, respectively. By maturity, both the Pn and relative chlorophyll values in the TNF treatments declined to lower levels. CRF_225_ had Pn and relative chlorophyll values 14.21% and 9.36% higher than CRF_135_, respectively, whereas compared to TNF_225_, CRF_225_ increased the Pn and relative chlorophyll values by 30.58% and 7.15%, respectively.

**Table 6:** ANOVA for the main and interaction effects of fertilizer type (T) and rate (R) on Net photosynthetic rate (Pn, μmol CO_2_/m^2^/s) under different nitrogen fertilizer types (CRF, TNF) and application rates (135, 225, 315 kg/ha) across 2019–2021.

Stage	ANOVA	2019	2020	2021	Total
Seeding	T	0.41ns	6.23*	3.03ns	3.44ns
	R	56.01**	76.65**	61.15**	162.73**
	T×R	3.93*	7.93**	4.05*	4.04*
Budding	T	0.35ns	0.82ns	0.50ns	1.51ns
	R	9.11**	29.49**	24.65**	55.97**
	T×R	0.76ns	7.68**	6.04**	9.29**
Flowering	T	303.65**	276.58**	272.31**	708.21**
	R	51.46**	59.06**	54.59**	135.64**
	T×R	1.42ns	9.16**	7.30**	13.08**
Maturity	T	517.35**	969.76**	534.35**	1157.83**
	R	62.28**	115.76**	98.13**	158.30**
	T×R	1.99ns	4.35*	3.19*	5.09**

*p ≤ 0.05, **p ≤ 0.01.

**Table 7:** ANOVA for the main and interaction effects of fertilizer type (T) and rate (R) on relative chlorophyll values under different nitrogen fertilizer types (CRF, TNF) and application rates (135, 225, 315 kg/ha) across 2019–2021.

Stage	ANOVA	2019	2020	2021	Total
Seeding	T	31.77**	43.37**	33.43**	107.32**
	R	12.82**	23.34**	18.09**	53.14**
	T×R	0.15ns	3.88*	0.76ns	1.59ns
Budding	T	21.85**	6.23**	7.37**	26.92**
	R	11.63**	4.19*	3.04ns	15.18**
	T×R	0.47ns	0.16ns	0.01ns	0.04ns
Flowering	T	6.57**	6.53**	5.10**	17.43**
	R	8.42**	6.90*	6.44*	21.05**
	T×R	0.58ns	0.06ns	0.72ns	0.66ns
Maturity	T	18.62**	20.04**	6.72**	29.10**
	R	7.33**	3.81ns	12.57**	16.11**
	T×R	0.20ns	0.32ns	0.22ns	0.12ns

**p ≤ 0.01.

**Figure 8:**
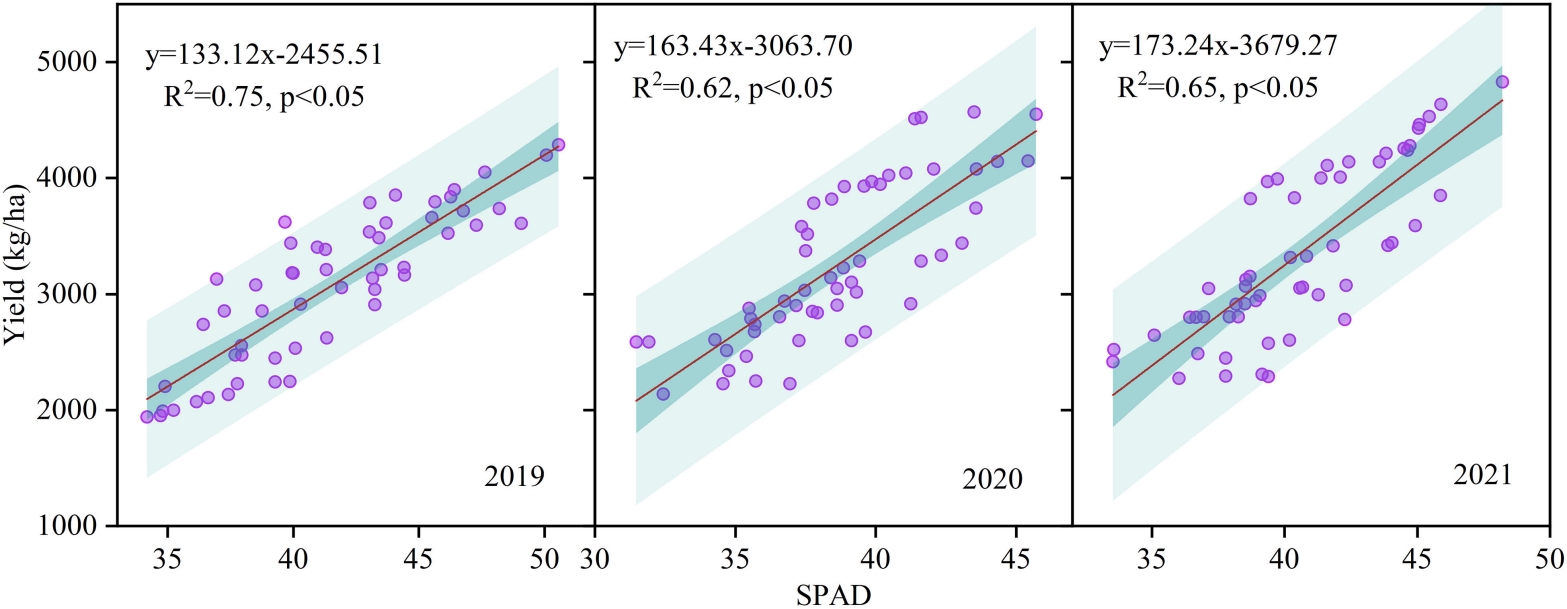
Correlation between grain yield and relative chlorophyll values under different nitrogen fertilizer types (CRF, TNF) and application rates (135, 225, 315 kg/ha) across 2019–2021. Relative chlorophyll values (SPAD readings).

Between 2019 and 2021, sunflower yield demonstrated a strong positive association with relative chlorophyll values across various nitrogen treatments and fertilizer regimes (**Figure 8**), with R^2^ ranging from 0.62 to 0.75, all statistically significant at p < 0.05.

### 3.5 Correlation analysis among soil nitrogen, root traits, photosynthesis, and yield

**Figure 9:**
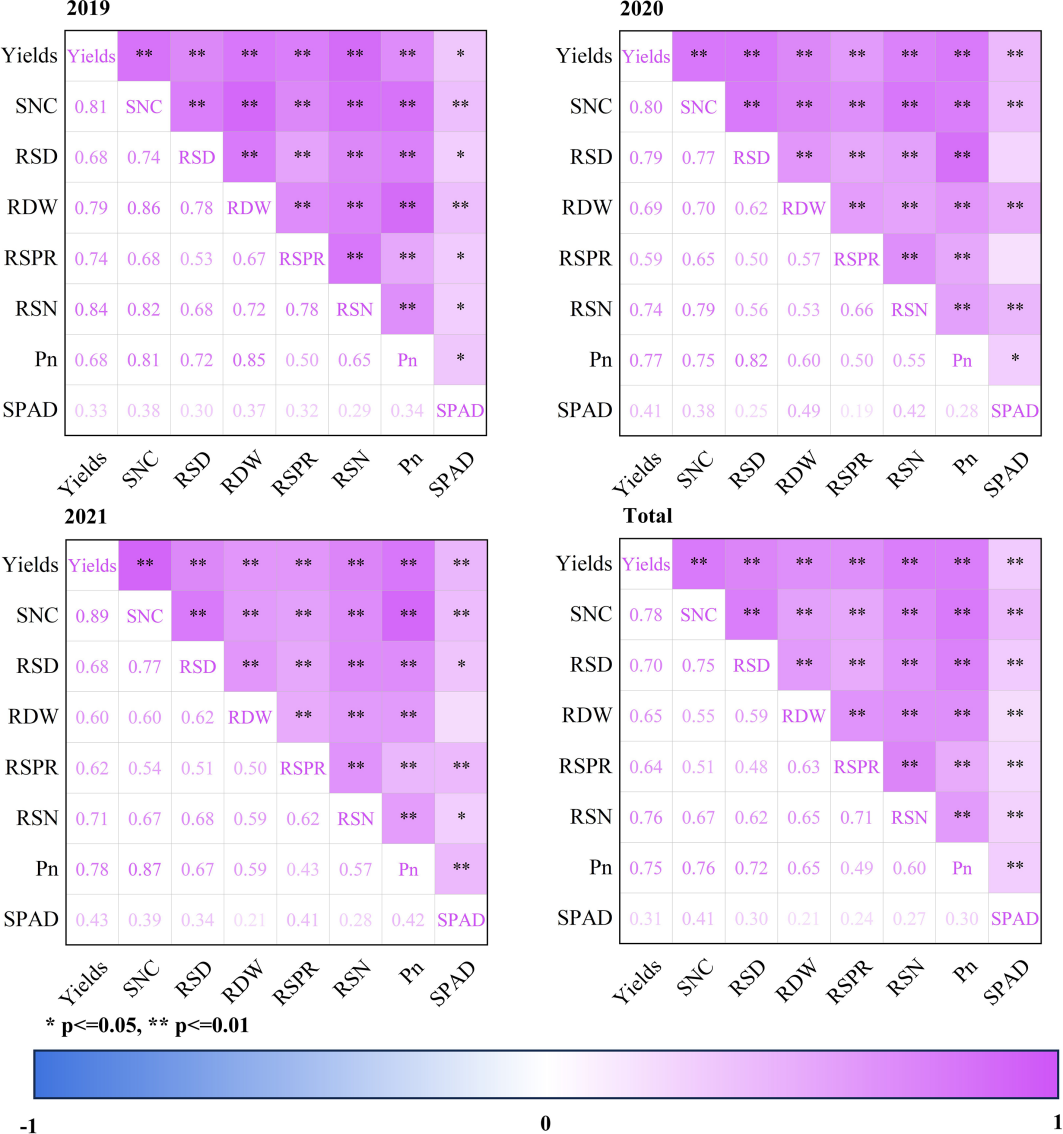
Correlation matrices showing the relationships among grain yield (Yields), soil nitrate concentration (SNC), root surface area density (RSD), root dry weight (RDW), root sap production rate (RSPR), root sap nitrate concentration (RSN), net photosynthetic rate (Pn), and chlorophyll content (SPAD) under different fertilization treatments in 2019, 2020, 2021, and the combined dataset (Total). Color intensity represents correlation strength, with p < 0.05 and p < 0.01 indicating significant and highly significant correlations, respectively. *p ≤ 0.05, **p ≤ 0.01.

To further clarify the interrelationships among key variables, this study conducted annual and overall correlation analyses (2019–2021) for SNC, RSD, RDW, RSPR, RSN, Pn, SPAD, and yield (**Figure 9**). The results showed that yield was consistently and positively correlated with SNC, RSD, RDW, RSN, and Pn across all three years (p<0.05 or p<0.01), with SNC exhibiting the strongest correlation with yield (r=0.78–0.89), indicating that soil nitrogen supply is a primary limiting factor for sunflower productivity.

Among root traits, both RSD and RDW showed significant positive correlations with yield (r=0.60–0.79), and were also strongly associated with SNC and RSN. This suggests that CRF maintains higher nitrogen availability in the root zone, thereby promoting root growth and nitrogen acquisition capacity. RSPR—an indicator of root metabolic activity—also displayed stable positive correlations with RSN, Pn, and yield, further supporting the mechanistic pathway of “enhanced root activity, increased nitrogen uptake, higher yield.” Among leaf physiological parameters, Pn had a stronger correlation with yield than SPAD, indicating that photosynthetic capacity more directly reflects the contribution of nitrogen management to yield formation than chlorophyll concentration alone.

The combined “Total” correlation analysis across the three years revealed a more stable correlation structure that was largely consistent with the annual results, though with slightly lower coefficient values. This reduction may be attributed to inter-annual variability in weather conditions, differences in growth duration, and seasonal variations in nitrogen transformation rates. Nonetheless, the overall patterns clearly indicate that CRF enhances soil nitrogen availability, promotes root development and nitrogen uptake efficiency, and consequently improves photosynthetic performance and final yield.

## 4 Discussion

### 4.1 Influence of various fertilization strategies on the sunflower root system

In this study, CRF application led to notable improvements in both RSD and RDW, with CRF_315_ reaching peak values of 3.53 cm^2^/cm^3^ and 14.70 g/plant at the flowering stage (**Table 2**). These findings indicate that CRF improves SNC, fosters better root architecture, and facilitates more efficient nitrogen uptake by crops (Olmo et al., 2015; Wei et al., 2020), particularly under nutrient-limited conditions or reduced nitrogen input. Due to its controlled nutrient release characteristics, CRF enables a more gradual and reliable nitrogen supply, which contributes to improved root development (Gouda et al., 2024; Su et al., 2024). In this experiment, RSD and RDW under CRF treatment were enhanced by 13.19% and 9.77%, respectively, when compared to the TNF group at the flowering stage. CRF enhanced root traits can be largely attributed to its sustained and steady nitrogen release pattern, which contrasts sharply with the rapid-release behavior of TNF (Li et al., 2021; Wang et al., 2025). The gradual nutrient supply moderates fluctuations in surface-soil nitrate concentration and slows nitrate downward movement after irrigation, thereby maintaining higher nitrogen availability in the root zone and promoting continuous fine-root proliferation and root-hair formation (Ren et al., 2025; Zhao et al., 2025). In addition, a stable N supply has been reported to enhance the activities of root-vigor–related enzymes and improve rhizosphere pH buffering capacity, jointly increasing the efficiency of water and nutrient uptake (Ma et al., 2023b; Zhang et al., 2021b). These mechanisms likely explain the observed increases in root surface area density and root dry weight under CRF in this study.

The data revealed that sunflowers receiving CRF treatment exhibited a 5.24% improvement in water and nutrient transport efficiency compared to those treated with TNF (**Figure 6**), supporting the interpretation that RSPR reliably reflects root vigor. This observation aligns with earlier studies, which demonstrated that RSPR is indicative of the physiological performance of root systems (Ansari et al., 2004; Liang et al., 2020). Moreover, the elevated RSN levels further validated CRF’s benefit in improving nitrogen uptake efficiency, as RSN under CRF conditions was 21.35% higher than with TNF. These results are consistent with those of (Gao et al., 2021), indicating that CRF not only contributes to enhanced root development but also facilitates more effective nitrogen absorption and internal transport—key processes for efficient soil nitrogen utilization under CRF regimes.

In this study, sunflowers treated with CRF exhibited more robust root development because the slow-release properties of CRF ensured a consistent nutrient supply, facilitating deeper root penetration into the soil (Govil et al., 2024; Ma et al., 2025; Zheng et al., 2023). Improved root system development under CRF treatment enabled plants to more effectively absorb moisture and nutrients from deeper soil horizons, thereby alleviating stress associated with suboptimal soil environments (Hou et al., 2024; Mikula et al., 2020). CRF application was shown to not only foster favorable conditions for root development, but also to contribute to increased crop yield potential by enhancing the efficiency of nutrient absorption and internal redistribution (Deng et al., 2025). These results underscore the critical role of fertilizer formulation and deployment strategy in shaping modern agricultural performance, especially in the context of adapting to climate-related stresses and mounting ecological constraints.

In addition to the overall differences among fertilizer types, the temporal dynamics of soil nitrate and root traits further explain the contrasting root development patterns observed between CRF and TNF. Soil nitrate under TNF increased sharply after basal application and topdressing but declined rapidly following each irrigation event, resulting in fluctuating N availability throughout the season. In contrast, CRF maintained a more stable nitrate supply over time (**Figure 2**), particularly during the bud and flowering stages when sunflower N demand peaks. Correspondingly, root traits followed similar temporal patterns: early-stage differences in RSD and RDW were small, but as the season progressed, CRF increasingly promoted deeper primary-root extension and sustained fine-root proliferation, whereas TNF-induced rapid early N release favored lateral root growth concentrated in shallower layers (**Figure 4**). These synchronized temporal trends highlight that the superior root architecture under CRF is closely linked to its gradual N release pattern, which better matches crop demand and reduces the mismatch caused by the rapid depletion of nitrate under TNF.

### 4.2 Effects of different fertilizer treatments on photosynthesis of sunflower

This study suggested that CRF treatment significantly enhanced the Pn of sunflower leaves at all N application levels, which is consistent with previous research. Studies have indicated that compared to TNF, CRF treatment increases leaf Pn, stomatal conductance, fluorescence intensity, transpiration rate, and dark respiration efficiency (Ma et al., 2023a). In this study, CRF treatment improved sunflower leaf Pn by 30.58% compared with TNF (**Figure 6**), which could be attributed to its controlled-release properties, which ensured a steady and balanced nitrogen supply throughout the growth period (ME Trenkel, 2021; Zheng et al., 2016). In this study, compared to TNF, CRF treatment likely provided a more consistent N supply during the later growth stages, sustaining the enzyme activity in the leaves and significantly boosting the activity of enzymes such as RuBPCase and RuBisCO, thereby improving the sunflower photosynthetic efficiency.

Research has indicated that CRF treatment can significantly increase the chlorophyll content in sunflowers, improve photosynthetic efficiency, and potentially enhance adaptability to environmental stresses (Ahmad et al., 2021; Ameen et al., 2024). Through a detailed analysis, this study clarified the mechanisms by which different fertilizer treatments affect sunflower photosynthesis, providing a scientific basis for optimizing agricultural production strategies with significant implications for advancing agricultural technology and practice. Additionally, further exploration is needed to understand how CRF can influence the rhizosphere environment through its physical and chemical properties, which may in turn affect photosynthetic mechanisms in chloroplasts. CRF can improve nutrient absorption and utilization by optimizing rhizosphere pH and microbial activity, indirectly influencing the activity and expression of photosynthesis-related enzymes.

### 4.3 Impact of different fertilization treatments on sunflower yield

In this study, sunflower yield was significantly influenced by fertilization type and N level (**Figure 3**). The CRF treatments demonstrated a clear advantage in enhancing yield, particularly at higher N levels, with CRF_225_ and CRF_315_ yielding significantly higher yields than the other treatments. This could be attributed to the continuous and uniform N supply provided by CRF, which improved NUE and increased the overall yield. Research has shown that CRF application can promote fine root growth, enhance NUE by 37.73%, and boost yield by an average of 21.35% (Zhang et al., 2021b). The positive correlation between photosynthesis and yield (**Figure 8**) suggests that improved photosynthetic efficiency can directly increase biomass accumulation, which is a key factor in yield enhancement (Feng et al., 2024; Qi et al., 2020). By increasing chlorophyll content and photosynthetic efficiency, CRF treatments can enable sunflowers to convert light energy into chemical energy more effectively, leading to greater yield accumulation throughout the growing season (Bassham, 1977; Sanchez-Bragado et al., 2020). Additionally, CRF application can mitigate environmental concerns associated with excessive nitrogen application and poor management, such as groundwater contamination and greenhouse gas emissions (Kassem et al., 2024; Xu et al., 2023). By improving NUE, CRF not only enhances the economic benefits of agricultural production but also aligns with sustainable development goals. Although temperature and rainfall varied among years (**Figure 1**), all treatments within the same year experienced identical conditions, so climate did not cause treatment differences. The consistent results across years further confirm that the yield advantages of CRF over TNF are robust and not confounded by climatic variation. The improvement in NUE observed under CRF treatments in this study also carries important environmental implications. Because CRF releases nitrogen more gradually and in closer synchrony with crop N demand (Zhang et al., 2024), it can substantially reduce the risk of nitrate leaching under irrigation (Wu et al., 2021b) while also minimizing surface-soil nitrogen accumulation that may otherwise lead to volatilization or denitrification losses (Ahmad et al., 2025). Numerous studies have shown that higher NUE is strongly associated with lower residual soil nitrate, reduced NH_3_ volatilization, and decreased N_2_O emissions, indicating that promoting CRF use in irrigated regions could help mitigate agricultural nitrogen pollution and greenhouse gas emissions (LÜ et al., 2023; ME Trenkel, 2021).

These results indicate that optimizing fertilization strategies, particularly through the utilization of CRF, can stimulate sunflower root growth and enhance the ability of plants to absorb water and nutrients from the soil (**Table 3**). This approach also increased chlorophyll content, improved photosynthetic efficiency, and ultimately enhanced both yield and NUE, making it essential for developing efficient and environmentally friendly agricultural production systems. Although this study provides clear evidence that CRF improves soil–root–plant nitrogen synchrony and enhances yield under arid irrigated conditions, several limitations should be acknowledged. First, the experiment was conducted on a single soil type typical of the Hetao Irrigation District, and soil texture and organic matter content may influence nitrogen mineralization, nitrate mobility, and CRF release behavior. Second, only one sunflower cultivar (SH361) was evaluated, and varietal differences in root architecture, nutrient uptake capacity, and stress tolerance may affect responses to CRF and nitrogen rate. Third, the study was carried out under furrow irrigation, whereas drip or sprinkler irrigation systems may alter nitrogen transport and CRF effectiveness. These factors limit the generalizability of the results to regions with similar soil properties, climatic conditions, and management systems. Future multi-site and multi-cultivar trials across different soil textures and irrigation methods will be essential to validate the broader applicability of the findings.

Practical implications of this study highlight that CRF can serve as an effective strategy for improving nitrogen use efficiency and reducing the risk of nitrate accumulation under arid irrigated conditions. For field management, applying CRF at moderate nitrogen rates (225 kg N/ha) may offer the best balance between yield performance and input cost, thereby enhancing both agronomic and economic returns. CRF may also reduce labor and application frequency, contributing to lower operational costs. In terms of future research, long-term trials are required to evaluate the sustainability of repeated CRF use, including its effects on soil structure, nutrient cycling, and residual nitrate accumulation. Moreover, the interactions between CRF, rhizosphere microbial communities, and soil biochemical processes remain poorly understood and warrant further investigation. Climate variability—including changes in temperature, evapotranspiration, and irrigation scheduling—may also influence CRF release patterns and crop responses; therefore, integrating CRF performance into climate-adaptive irrigation and fertilization models will be essential for optimizing fertilizer strategies in arid and semi-arid regions. Overall, these findings reinforce the importance of integrating CRF-based nitrogen management into arid irrigated cropping systems to achieve both high productivity and environmental sustainability.

## 5 Conclusions

This study systematically evaluated the effects of different fertilizer types and application rates on sunflower growth, with a particular focus on the role of CRF on yield, root development, photosynthetic efficiency, and NUE. The results indicated that owing to its sustained-release characteristics, CRF significantly optimized RLD and RDW, enhancing the ability of roots to absorb water and nutrients. At higher N levels (CRF_225_ and CRF_315_), CRF played a crucial role in promoting root health, expansion, and plant adaptation to environmental stress. Additionally, CRF treatment significantly increased the net photosynthetic efficiency and chlorophyll content across all N levels, suggesting that a stable N supply improved photosynthetic conditions, leading to more efficient energy conversion and biomass accumulation. In terms of yield and NUE, the CRF treatments not only increased the crop yield but also improved the N conversion efficiency by reducing losses, demonstrating the evident advantages in enhancing NUE. These findings provide valuable guidance for agricultural producers in selecting fertilizer types and application strategies. Future research should further explore the potential of CRF across different agricultural ecosystems, considering the variations in crop varieties, soil conditions, and climate change impacts on N responses to enhance the efficiency and sustainability of N management. Additionally, optimizing fertilizer formulations and application strategies should be prioritized to simultaneously maximize crop yield and minimize environmental impact.

## Data Availability

The original contributions presented in the study are included in the article/supplementary material. Further inquiries can be directed to the corresponding author.
